# *Equisetum arvense* (common horsetail) modulates the function of inflammatory immunocompetent cells

**DOI:** 10.1186/1472-6882-14-283

**Published:** 2014-08-04

**Authors:** Carsten Gründemann, Karin Lengen, Barbara Sauer, Manuel Garcia-Käufer, Martin Zehl, Roman Huber

**Affiliations:** Center for Complementary Medicine, Department of Environmental Health Sciences, Medical Center, University of Freiburg, Breisacherstr. 115B, 79106 Freiburg, Germany; Department of Pharmacognosy, University of Vienna, Althanstrasse 14, A-1090 Vienna, Austria

**Keywords:** *Equisetum arvense*, Equisetopsida, Horsetail, Inflammation, Lymphocytes, Immunosuppression, Anthroposophical Medicine

## Abstract

**Background:**

In Europe, extracts of *Equisetum arvense* (common horsetail) have a long tradition in the treatment of inflammatory disorders. To understand the molecular basis for its use, we investigated the immunomodulatory capacity of a standardized commercially available common horsetail extract on human primary lymphocyte function *in vitro.*

**Methods:**

The standardized extract of *Equisetum arvense* was phytochemically characterized. Effects on proliferation, viability and activity of mitogen-activated human lymphocytes were assessed in comparison to cyclosporine A using annexin V/propidium iodide staining assays and flow cytometry-based surface receptor characterization, respectively. Intracellular levels of effector molecules (IL-2, IFN-γ and TNF-α) were analyzed with cytokine assays.

**Results:**

T cell proliferation was inhibited dose dependently by the *Equisetum* extract without induction of apoptosis or necrosis. This effect was mediated through inhibition of lymphocyte activation, specifically by diminishing CD69 and IL-2 surface receptor expression and intracellular IL-2 production. Furthermore, treatment with *Equisetum arvense* inhibited effector functions, as indicated by reduced production of IFN-γ and TNF-α.

**Conclusions:**

The data indicate that the used extract of *Equisetum arvense* interferes with the polyfunctionality of immunocompetent cells thereby providing an anti-inflammatory mode-of-action.

**Electronic supplementary material:**

The online version of this article (doi:10.1186/1472-6882-14-283) contains supplementary material, which is available to authorized users.

## Background

*Equisetum arvense* (common horsetail, field horsetail, or giant horsetail) belongs to the Equisetopsida family and is native to the Arctic and temperate regions of the northern hemisphere, particularly Europe [1]. Its traditional use as an herbal remedy is documented in several handbooks of phytotherapy [2, 3] and continues to be popular in complementary medicine, especially Anthroposophical Medicine [4]. Among the species of this genus, only *Equisetum arvense* “Equiseti herba”) is listed in the German commission E Monograph (phytotherapy and herbal substances) of the German Federal Institute for Drugs and Medical Devices [5] as well as in the European Pharmacopoeia [6]. The putative medicinal properties are supported by a number of studies, which found (I) hepatoprotective [7], (II) diuretic [8], (III) anti-bacterial [9, 10] or (IV) antioxidant effects [11–14]. Furthermore, (V) anti-inflammatory properties for the treatment of wounds or inflammatory diseases such as arthritis have been described [15, 16]. However, to date, there are no studies on the impact of *Equisetum arvense* extracts on lymphocytes involved in inflammatory immune processes. Lymphocytes are the body’s second line of defence and T cells are actively recruited to sites of inflammation where they maintain and activate fibroblasts or bystander dendritic cells and macrophages, transforming them into tissue-destructive effector cells [17]. T lymphocytes are the dominant cells in inflammatory immune diseases [18, 19], and their proliferation and mediator release (IFN-γ, TNF-α) are targets for modern therapies [20]. Immunosuppressants such as glucocorticoids [21] or calcineurin inhibitors, e.g. cyclosporine A, which specifically down-regulate the immune system [20], are the established treatment of choice. Despite the availability of effective conventional medications, approximately 60-90% of patients with inflammatory immune disorders resort to alternative or complementary therapies to avoid side effects [22]. One such therapy involves use of *Equisetum arvense*. Because we were interested in exploring the “active principle”, we phytochemically characterized a standardized, commercially available extract of *Equisetum arvense* and defined its immunosuppressive capacity on cell proliferation, activation status and effector functions using mitogen-activated human lymphocytes.

## Methods

### Plant material

*Equisetum arvense* (Equisetopsida), ethanol. Decoctum (=Equisetum: D1; Weleda AG, Schwäbisch Gmünd, Germany) was used for the experiments. The extract is manufactured according to method no. 19f of the German Homeopathic Pharmacopoeia [23]. The *Equisetum arvense* was of European origin and came from wild harvests. The fresh plant material was kept refrigerated and sent to Weleda Naturals GmbH, Schwäbisch Gmünd, Germany, where the identity of the plant material was analyzed by botanic specialists using macroscopic and microscopic methods as well as comparison with depositions of specified botanical reference stocks. The plant material was dried, homogenized and a decoct was produced by boiling one part dried plant material with nine parts ethanol for 4 h. The absolute ethanol concentration of the preparation used was 36 Vol.-%. We used the same concentration of ethanol for vehicle control experiments and clearly demonstrated that these amounts have no influence on the behaviour of the cells. After cooling down to room temperature, the decoct was squeezed out mechanically, filtered sterile and filled into 50 mL bottles. Bottles from sale stocks were sent to our laboratory in Freiburg, Germany, where the experiments were performed. For each experiment, a fresh frozen aliquot of the *Equisetum arvense* extract was used, and concentrations of 0.05, 0.1, 0.2, 0.4 and 0.8 μg/mL were tested.

### Phytochemical analysis of the *Equisetum arvense*extract

LC-MS analyses were performed on an UltiMate 3000 RSLC-series system (Dionex, Germering, Germany) coupled to a 3D quadrupole ion trap mass spectrometer equipped with an orthogonal ESI source (HCT, Bruker Daltonics, Bremen, Germany). HPLC separation was carried out on an Acclaim 120 C18, 2.1 × 150 mm, 3 μm column (Dionex) at 30°C using 0.1% aqueous formic acid and acetonitrile as mobile phase A and B, respectively. The flow rate was 0.5 mL/min and the following gradient program was used: 5% B (0 min), 5% B (2 min), 11% B (20 min), and 24% B (33 min), followed by a column cleaning and re-equilibration step. The eluent flow was split at a 1:4 ratio before the ESI ion source, which was operated as follows: capillary voltage: +3.5 kV, nebulizer: 26 psi (N_2_), dry gas flow: 9 L/min (N_2_), and dry temperature: 340°C. MS^2^, MS^3^, and MS^4^ spectra were obtained in an automated data-dependent acquisition mode (collision gas: He, isolation window: 4 Th, fragmentation amplitude: 1.0 V). 2 μL of the undiluted, centrifuged *Equisetum* extract were injected. Finally, two of the tentatively identified components were confirmed by comparison of the retention times, UV- and MS^n^-spectra with the reference compounds isoquercitrin (Carl Roth, Karlsruhe, Germany) and kaempferol-3-*O*-glucoside (Extrasynthese, Genay Cedex, France).

Quantification of the main flavonoid isoquercitrin (quercetin-3-*O*-glucoside) in the *Equisetum arvense* extract was performed by HPLC-DAD with external standard calibration, and the total flavonoid content was determined by a spectrophotometric assay adapted from the European Pharmacopoeia (Ph. Eur.) Monograph on Equiseti herba [6] (for details see Additional file 1).

### Ethics statement

Patients gave their written consent for giving blood for scientific research.

All experiments conducted on human material were approved by the Ethics committee of the University of Freiburg (55/14).

### Preparation and cultivation of human peripheral lymphocytes

Human peripheral lymphocytes (PBMC) were isolated from the blood of healthy adult donors obtained from the Blood Transfusion Centre (Medical Center, University of Freiburg, Germany). Venous blood was centrifuged on a LymphoPrep™ gradient (density: 1.077 g/cm^3^, 20 min, 500 × g, 20°C; Progen, Heidelberg, Germany). Cells were washed twice in medium, and cell viability as well as concentration was determined using the trypan blue exclusion test. Cells were cultured in RPMI 1640 medium supplemented with 10% heat-inactivated fetal calf serum (PAA, Pasching, Austria), 2 mM L-glutamine, 100 U/mL penicillin and 100 U/mL streptomycin (all from Life Technologies, Paisley, UK). The cells were cultured at 37°C in a humidified incubator with 5% CO_2_/95% air atmosphere.

### Activation and treatment of lymphocytes and T-cells

PBMC (10^5^) were stimulated with phytohemagglutinin-L (PHA-L; 10 μg/mL; Roche Diagnostics, Mannheim, Germany) or lipopolysaccharide (LPS; 1 μg/mL; Sigma-Aldrich, Taufkirchen, Germany) in the presence of control agents cyclosporine A (CsA; 10 μg/mL obtained by dilution of Sandimmun® 50 mg/mL, Novartis Pharma GmbH, Nürnberg, Germany), camptothecin (CPT; 30 μg/mL; Tocris, Eching, Germany) and Triton-X 100 (0.5%; Carl Roth, Karlsruhe, Germany), or different concentrations of *Equisetum arvense* extract. After cultivation, the cells were assessed in bioassays as described in the text.

### Cell division tracking using CFSE

PBMC were harvested and washed twice in cold PBS before they were resuspended in PBS at a concentration of 5 × 10^6^ cells/mL. CFSE (carboxyfluorescein diacetate succinimidyl ester, 5 mM; Sigma, Taufkirchen, Germany) was added in 1/1000 dilution and the PBMC were incubated for 10 min at 37°C. The staining reaction was stopped by washing twice with complete medium. Afterwards, the cell division progress was analyzed using flow cytometry.

### Determination of apoptosis and necrosis using annexin V and propidium iodide staining

PBMC were cultured in the presence of *Equisetum arvense* extract for 24 or 48 h, respectively, and the levels of apoptosis and necrosis were determined using Annexin V-FITC apoptosis detection kit (eBioscience, Frankfurt, Germany or BD Bioscience, Heidelberg, Germany) according to the manufacturer’s instructions. After annexin V staining, propidium iodide solution (PI; eBioscience or BD Bioscience) was added and the cells were incubated in the dark, followed by a flow cytometric analysis to determine the amount of apoptosis and necrosis. We used CPT (100 μM) and Triton-X 100 (0.5%) as positive controls for apoptosis and necrosis, respectively.

### CD69 activation marker and IL-2 surface receptor analysis

Cultured cells (24 or 48 h) were washed with PBS and stained with FITC-labeled anti-human CD69 and PE-labeled anti-human CD25 mAbs (both eBioscience, Frankfurt, Germany) for 15 min at 4°C. Next, the cells were washed twice with PBS, resuspended and transferred into FACS vials. The expression of CD69 and IL-2 surface receptor α-chain CD25 was measured by FACS analysis using a FACSCalibur instrument.

### Determination of cytokine production

Cells were treated for 36 h and were restimulated with PMA (50 ng/mL) and ionomycin (500 ng/mL) (both from Sigma-Aldrich, Taufkirchen, Germany) for additional 6 h. Cytokines were measured using intracellular cytokine analysis. For this purpose, the cells were surface-stained with anti-human CD8 mAbs (eBioscience), fixed, and permeabilized using 4% paraformaldehyde (Sigma-Aldrich) and Perm/Wash solution (Becton Dickinson, Franklin Lakes, NJ), followed by staining with PE-conjugated anti-human IFN-γ mAb or anti-human TNF-α mAb (both from eBioscience), respectively. Samples were analyzed with a BD FACSCalibur flow cytometer using BD CellQuest Pro Software.

### Analysis of data

For statistical analysis, data were processed with Microsoft Excel and IBM SPSS software 20.0. Values are presented as mean ± SD for the indicated number of independent experiments. Statistical significance was determined by one-way ANOVA followed by Dunnett’s post hoc pairwise comparisons. For estimation of the inhibitory concentration (IC50), a probit regression model for the dose-response curve was used and data were processed by ToxRat® software. The asterisks represent significant differences from controls (*P < 0.05, **P < 0.01, ***P < 0.001).

## Results

### Phytochemical analysis of the *Equisetum arvense*extract

**Figure 1:**
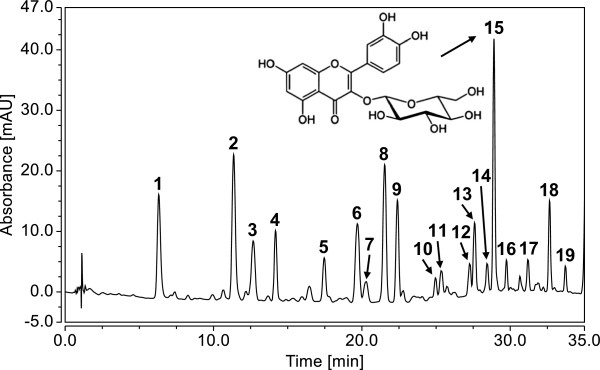
**Secondary plant metabolites identified in the** ***Equisetum arvense*** **extract.** The herbal preparation was analysed by LC-MS and the tentatively identified compounds are labelled in the given HPLC chromatogram showing the DAD response at 340 ± 2 nm: monocaffeoyl-tartaric acid isomer (1 and 2), monoferuloyl-tartaric acid isomer (3 and 7), kaempferol-3-*O*-sophoroside-7-*O*-glucoside (4), quercetin-3,7-di-*O*-glucoside (5), caffeoyl-malic acid (6), kaempferol-3,7-di-*O*-glucoside (8), kaempferol-3-*O*-rutinoside-7-*O*-glucoside (9), quercetin-3-*O*-sophoroside (10), 4-coumaric acid (11), kaempferol-3-*O*-sophoroside (12), caffeic acid methyl ester (13), protogenkwanin-4’-*O*-glucoside (14), quercetin-3-*O*-glucoside (15), apigenin-*O*-glucoside (16), kaempferol-3-*O*-glucoside (17), dicaffeoyl-tartaric acid (18), and genkwanin-*O*-glucoside (19). The corresponding MS-data are provided in Additional file 1: Table S1.

There is no clear correlation between traditional use of this herbal drug and any of its constituent in particular; however, the Ph. Eur. prescribes a minimum of 0.3% of total flavonoids expressed as isoquercitrin in the dried drug [6]. Consequently, we also focused our phytochemical analysis of the *Equisetum arvense* extract on flavonoids and other polar phenolics. First, an HPLC method was developed to separate the phenolic compounds in the extract (Figure 1), which were then tentatively identified by LC-MS with an ESI-ion trap instrument (Additional file 1: Table S1). Major phenolic constituents of the *Equisetum arvense* extract were found to be various mono-, di-, and triglycosides of kaempferol, quercetin, apigenin, genkwanin and protogenkwanin, as well as some hydroxycinnamic acid derivatives, most prominently mono- and dicaffeoyl-tartaric acid. The presence of quercetin-3-*O*-glucoside (isoquercitrin) and kaempferol-3-*O*-glucoside was confirmed by comparison of the retention times, UV- and MS^n^-spectra with reference compounds. Finally, we determined the total flavonoid content of the *Equisetum arvense* extract by a spectrophotometric assay and the concentration of isoquercitrin by an HPLC-DAD method (see Additional file 1).

### *Equisetum arvense*decreased proliferation of mitogen-activated human lymphocytes

**Figure 2:**
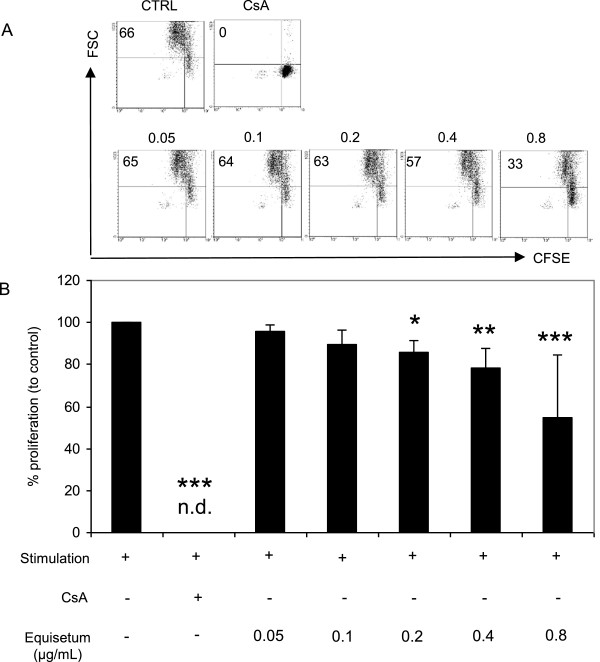
**Effects of** ***Equisetum arvense*** **on proliferation of primary human lymphocytes.** CFSE-labeled PHA-L (10 μg/mL)-activated human PBMC (10^5^) were treated with medium, CsA (10 μg/mL) or increasing concentrations of *Equisetum arvense* (0.05-0.8 μg/mL). Cell division analysis was assessed by flow cytometry and depicted as representative dot plots **(A)** for bulk lymphocytes. Results are summarized in **(B)** and data are presented as mean ± SD of five independent experiments and donors. n.d. = no detection. The asterisks (*P < 0.05, **P < 0.01, ***P < 0.001) represent significant differences from activated untreated controls (CTRL =100%).

In order to analyze the immunomodulatory activity of *Equisetum arvense*, we evaluated the effects on cell growth and performed proliferation assays with increasing concentrations of the *Equisetum* extract (0.05-0.8 μg/mL) using CFSE-labeled mitogen (PHA-L)-activated lymphocytes (Figure 2). The CFSE dye is inherited from daughter cells after cell division and each dividing cell loses fluorescence intensity without affecting cell viability. The flow cytometry analysis demonstrated that the proliferation of CFSE^+^ mitogen-activated lymphocytes was strongly inhibited to levels of no detection in the presence of the positive control cyclosporine A (CsA) compared to stimulated cells alone (=100%). The presence of *Equisetum* concentration-dependently (0.05 μg/mL: 95% ± 3.2; 0.1 μg/mL: 89% ± 6.6; 0.2 μg/mL: 86% ± 5.1; 0.4 μg/mL: 78% ± 9.8; 0.8 μg/mL: 54% ± 29.8) decreased the proliferative capacity of human immunocompetent cells compared to stimulated cells alone (=100%) with an IC50 value of 1.09 μg/mL.

### *Equisetum arvense*-mediated inhibition of proliferation is not mediated through apoptosis or necrosis induction

**Figure 3:**
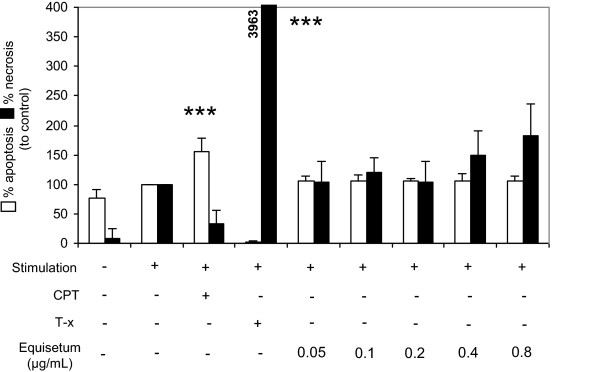
**Effects of** ***Equisetum arvense*** **on apoptosis and necrosis induction in primary human lymphocytes.** 10^5^ PBMC were cultured for 72 h and left non-stimulated or PHA-L-activated (10 μg/mL) in the presence of medium (CTRL =100%) or in the presence of different *Equisetum arvense* concentrations (0.05-0.8 μg/mL). Next, the cells were stained with annexin V and propidium iodide and analyzed using flow cytometry. CPT (100 μM) and Triton-X100 (0.5%) were used as apoptosis and necrosis control, respectively. Results from analysis of apoptotic (white bars) and necrotic (black bars) cells are summarized and presented as mean ± SD of four independent experiments and donors. The asterisks represent significant differences from non-treated stimulated cells alone (***P < 0.001).

In a further step, we aimed to identify the mechanism behind the reduced cell proliferation observed, and quantified apoptotic and necrotic effects on activated lymphocytes in the presence of increasing concentrations (0.05-0.8 μg/mL) of the *Equisetum* extract. As positive controls for apoptosis and necrosis, we used camptothecin and Triton-X 100, respectively. As shown in Figure 3, positive controls significantly increased the amount of apoptotic^+^ (white bars; 156% ± 22) and necrotic^+^ (black bars; 3962% ± 725) cells. *Equisetum* concentrations had no significant influence on the induction of apoptosis or necrosis compared to stimulated untreated cells alone (=100%).

### *Equisetum arvense*influences cell proliferation capacity through partial inhibition of lymphocyte activation and IL-2 biology

**Figure 4:**
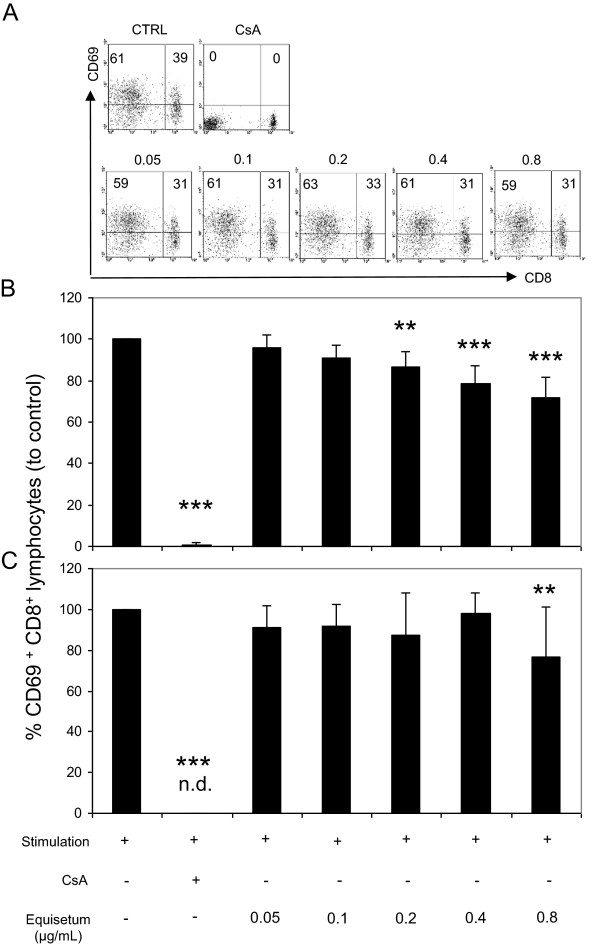
**Effects of** ***Equisetum arvense*** **on activation of primary human lymphocytes.** Stimulated CD8^+^ T cells were cultured in the presence of medium, CsA (10 μg/mL) or increasing concentrations (0.05-0.8 μg/mL) of the *Equisetum* extract. Cells were analyzed for CD69 expression using FITC-labeled anti-human mAbs. **(A)** Representative dot plots are shown and data were summarized from experiments at early (**B**; 24 h) or late (**C**; 48 h) time points from five independent experiments and donors. n.d. = no detection. The asterisks (**P < 0.01, ***P < 0.001) represent significant differences from activated untreated controls (CTRL =100%).

A reduced proliferative capacity could be mediated through inhibition of T cell activation. Figure 4 depicts the respective experiments and confirms that CsA-treated T-cells abrogate expression of CD69 completely (Figure 4B: 0.5% ± 1.1 and Figure 4C: no detection). *Equisetum* decreased the expression of CD69 (0.05 μg/mL: 95% ± 6.2; 0.1 μg/mL: 91% ± 6; 0.2 μg/mL: 86% ± 7.5; 0.4 μg/mL: 78% ± 8.9; 0.8 μg/mL: 71% ± 9.7) at early (Figure 4A and B) but not at late (Figure 4C) time points compared to controls (=100%) in a concentration-dependent manner, revealing that *Equisetum* exerts its anti-proliferative effect through inhibition of cell activation.

**Figure 5:**
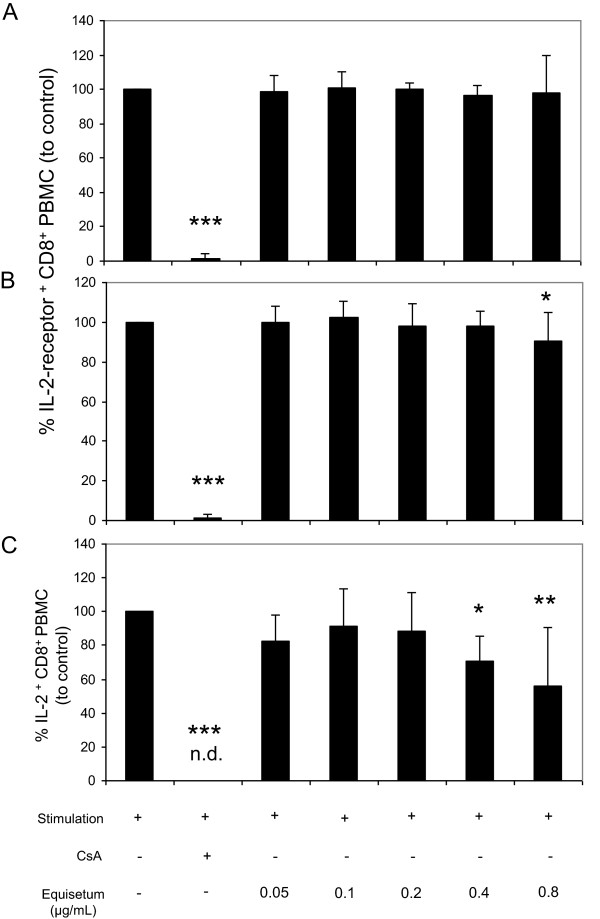
**Effects of** ***Equisetum arvense*** **on IL-2 biology of primary human lymphocytes.** Medium-, CsA or *Equisetum*-treated activated (PHA-L: 10 μg/mL) lymphocytes were surface-stained with anti-human CD25 mAbs and were analyzed at early (**A**; 24 h) or at late (**B**; 48 h) time points. For IL-2 production analysis, cells were treated for 36 h and were restimulated with PMA (50 ng/mL) and ionomycin (500 ng/mL) for additional 6 h followed by surface-staining with anti-human CD8 mAbs and intracellular cytokine assay with PE-conjugated anti-human IFN-γ mAbs or anti-human TNF-α mAbs **(C)**. Cells were analyzed using flow cytometry. Data were expressed and summarized as mean ± SD of five independent experiments and donors. The asterisks (*P < 0.05, **P < 0.01, ***P < 0.001) represent significant differences from activated untreated controls (CTRL =100%).

T cell proliferation is also initiated by activation and expression of the autocrine growth factor interleukin-2 (IL-2). This promotes interaction with the receptor CD25, which is up-regulated on the surface of activated T-cells [24] and by release of endogenous IL-2. Consequently, the influence of the *Equisetum arvense* extract on IL-2-receptor expression and IL-2 secretion was also analyzed (Figure 5). The results demonstrated that the *Equisetum* extract investigated has little impact on the expression of the IL-2 receptor at late time points at high (0.8 μg/mL: 90% ± 14) concentrations only (Figure 5B). It also inhibits production of IL-2 (Figure 5C; 0.4 μg/mL: 70% ± 14; 0.8 μg/mL: 55% ± 35) compared to controls (=100%). These data indicate that *Equisetum arvense* reduces proliferation of immunocompetent cells by inhibiting cell activation and IL-2 biology at more than one site.

### *Equisetum arvense*slightly affects effector function of activated human lymphocytes

**Figure 6:**
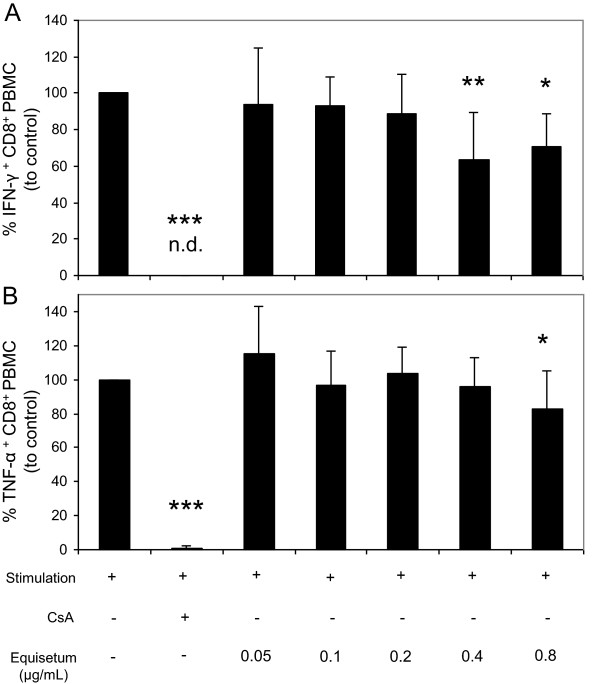
**Effects of** ***Equisetum arvense*** **on effector function of primary human lymphocytes.** Activated (PHA-L; 10 μg/mL) human PBMC (10^5^) were treated with medium, CsA (10 μg/mL) or increasing concentrations of *Equisetum arvense* (0.05-0.8 μg/mL) and were cultured for 36 h followed by a restimulation period with PMA (50 ng/mL) and ionomycin (500 ng/mL) for additional 6 h. Next, cells were surface-stained with anti-human CD8 mAbs and intracellular cytokines were detected using PE-conjugated anti-human IFN-γ or anti-human TNF-α mAbs. n.d. = no detection. The asterisks (*P < 0.05, **P < 0.01, ***P < 0.001) represent significant differences from activated untreated controls (CTRL =100%).

Consequently, it was of interest whether the *Equisetum* extract under investigation only inhibits proliferation of lymphocytes or also affects lymphocyte polyfunctionality, which would be directly related to changes in the production of interferon-gamma (IFN-γ) and tumor necrosis factor-alpha (TNF-α). The production of both mediators in the presence of CsA and *Equisetum* arvense was analyzed using a flow cytometry-based intracellular cytokine approach (Figure 6). The results showed a slight inhibition of the IFN-γ- (Figure 6A; 0.4 μg/mL: 63% ± 26; 0.8 μg/mL: 70% ± 18) as well as TNF-α- (Figure 6B; 0.8 μg/mL: 82% ± 22) production of activated T cells at high *Equisetum* concentrations, whereby the presence of CsA reduced secretion of these parameters to undetectable levels.

## Discussion

Extracts of *Equisetum arvense* (common horsetail) have a long tradition in the treatment of inflammatory disorders, for which reason we were interested in clarifying whether there is a rational basis for its use. To this end, we characterized a standardized common horsetail extract in cell-based assays using activated immunocompetent cells.

We found a concentration-dependent inhibition of mitogen-activated lymphocyte proliferation with an IC50 value of 1.09 μg/mL. Furthermore, our data indicate that the inhibition of proliferation induced by the *Equisetum arvense* extract was not mediated by apoptosis and necrosis. Immunosuppressive effects of *Equisetum arvense* have also been observed by other groups, however, their investigations were only done with human cancer cell lines [11, 25]. T-cell proliferation is a process initiated by ligation of the T-cell receptor to antigens that triggers a complex T-cell receptor signalling pathway. During this process, the human transmembrane C-Type lectin protein CD69 is expressed on the surface of activated T-cells [24]. CD69 is an early activation marker that is expressed at high amounts immediately after activation, and gene as well protein expression of this marker rapidly decreases 24 h after stimulation. We found a decrease in the activation status of stimulated T-cells after stimulation, whereby the effects of *Equisetum* are smaller than that of CsA. T-lymphocyte proliferation is further initiated by activation and expression of the autocrine growth factor interleukin-2 (IL-2), which promotes interaction with the receptor CD25 that is up-regulated on the surface of activated T-cells [24] and by release of endogenous IL-2. The expression of the IL-2 receptor was indeed slightly affected in the presence of *Equisetum*. Over time, however, inhibition was smaller than that of CsA, which specifically reduces the early activation state of lymphocytes due to suppression of the IL-2 receptor [26, 27]. The data further demonstrate that *Equisetum* reduces IL-2 cytokine production. Because IL-2 is pivotal for lymphocyte proliferation, inhibition of its production may at least partially explain the immunosuppressive effects of *Equisetum* in these experiments. In addition, a slight inhibition of IFN-γ- and TNF-α-production of activated T cells could be shown. Thus, we can conclude that the horsetail extract interferes with T-cell polyfunctionality, which results in diminished proliferation of immunocompetent cells.

The stems of *Equisetum arvense* contain high amounts of minerals, in particular silicic acid and silicates (5-8%), potassium and calcium, various flavonoids (0.2-0.9%) and phenolic acids, the phenolic petrosins onitin and oniti-9-*O*-glucoside, equisetumpyrone (only in the very early developmental stages), triterpenoids and phytosterols. They also contain low amounts of essential oil, the main constituents being hexahydrofarnesyl acetone, cis-geranyl acetone, thymol, and trans-phytol as well as a few other compounds [28, 29]. Especially, the considerable amounts of polyphenols and several compounds of this diverse group of phytochemicals have attracted interest in the past due to their presumptive physiological activities. However, there is no clear correlation between the traditional use of this herbal drug and any constituents in particular. The Ph. Eur. requires a minimum of 0.3% of total flavonoids expressed as isoquercitrin in the dried drug [6]. Consequently, we focused our phytochemical analysis of the investigated *Equisetum arvense* extract on flavonoids and other polar phenolics. The phenolic pattern is largely in agreement with a previous study on the variation of phenolics in different species of the genus *Equisetum* subgenus *Equisetum*, and is characteristic for the European chemotype of *E. arvense* [29, 30]. The main difference found in the qualitative composition to the study by Veit et al. [29] is that we did not detect significant amounts of malonylated flavonoid glycosides. Several of the numerous known components of the characterized *Equisetum* extract have been reported to possess anti-inflammatory activity [31–34]. We know from the literature, that given orally polyphenols (specifically flavanols, flavonols, flavanones, flavones) are absorbed by enterocytes and do not reach circulation [35]. Systemic effects of orally applied polyphenols therefore cannot be expected. Rather, topical treatment of the skin or mucosa would be more realistic to reach concentrations of *Equisetum* comparable to our *in vitro* experiments.

## Conclusions

The presented data indicate that the *Equisetum arvense* extract used interferes polyfunctionally with immunocompetent cells, thereby providing a potential mechanism that explains its traditional use in the treatment of inflammatory disorders. However, further studies should be conducted to prove its clinical potency.

## Electronic supplementary material

Additional file 1: **Quantification of isoquercitrin and total flavonoids in the** ***Equisetum arvense*** **extract.** (DOC 304 KB)
