# Supplementary material for: Equisetum arvense (common horsetail) modulates the function of inflammatory immunocompetent cells
Source: BMC Complement Altern Med. 2014 Aug 4;14:283. doi: 10.1186/1472-6882-14-283 (PMC4132922; doi:10.1186/1472-6882-14-283)
Supplement: Supplementary file 1 — Additional file 1: Quantification of isoquercitrin and total flavonoids in the Equisetum arvense extract. (DOC 304 KB) [file 12906_2014_1864_MOESM1_ESM.doc]

**Supplementary Material**

***Equisetum arvense* (Horsetail) modulates the function of inflammatory immunocompetent cells**

Carsten Gründemann^a*^, Karin Lengen^a^, Barbara Sauer^a^, Manuel Garcia-Käufer^a^, Martin Zehl^b^ and Roman Huber^a^

^a^Center for Complementary Medicine, Department of Environmental Health Sciences, University Medical Center Freiburg, Breisacherstr. 115B, 79106 Freiburg, Germany

^b^Department of Pharmacognosy, University of Vienna, Althanstrasse 14, A-1090 Vienna, Austria

***Correspondence:**Dr. Carsten Gründemann, PhD

Phone: +49 761 / 270-83170

Fax: +49 761 / 270-83230

Email: carsten.gruendemann@uniklinik-freiburg.de

**Quantification of isoquercitrin and total flavonoids in the *Equisetum arvense* extract**

Quantification of the main flavonoid isoquercitrin (quercetin-3-*O*-glucoside) in the *Equisetum arvense* extract was performed by HPLC-DAD with external standard calibration. The HPLC instrument and method were the same as for the LC-MS analyses. A stock solution of isoquercitrin (purity 99.97% by HPLC, Carl Roth) was prepared by dissolving 2.05 mg in 20.00 mL methanol. The five calibration solutions ranging from 1.025 to 102.5 µg/mL were obtained by stepwise dilution to 1:5, 1:10, 1:50, and 1:100 with methanol. The sample was diluted 1:5 with methanol and centrifuged. 2 µL of this sample and each standard solution were injected in quadruplicate. The chromatograms at 353 ± 2 nm were used to measure the peak areas. The concentration was determined using a five-point calibration giving a linear regression equation of y = 12362x + 2321 (R² = 0.999998), where x is the concentration of isoquercitrin in µg/mL. Thereby, the *Equisetum* extract was found to contain **43.1 ± 0.7 µg/mL isoquercitrin (n=4)**.

The total flavonoid content was determined by a spectrophotometric assay adapted from the European Pharmacopoeia (Ph. Eur.) monograph on Equiseti herba [6], whereby the only modification was that instead of 0.800 g of the powdered drug, 10.00 mL of the *Equisetum arvense* extract were dried under reduced pressure to obtain the stock solution. To the dried extract, 1 mL of a 5 g/L solution of hexamethylenetetramine, 20 mL of acetone, and 2 mL of hydrochloric acid (250 g/L HCl) were added. The mixture was boiled under a reflux condenser for 30 min and filtered through a plug of absorbent cotton. The residue together with the absorbent cotton were extracted twice with 20 mL of acetone, each, by boiling under a reflux condenser for 10 min. The combined extracts were filtered and diluted to 100.0 mL with acetone. 20.0 mL of this solution were diluted with 20 mL of water and partitioned with 15 mL, and then three times 10 mL of ethyl acetate. The combined ethyl acetate extracts were washed twice with 50 mL, each, of water, filtered over 10 g of anhydrous sodium sulphate, and diluted to 50.0 mL with ethyl acetate.

The test solution was prepared by addition of 1 mL of aluminium chloride reagent (2.0 g AlCl_3_ dissolved in 100 mL of a 5 % (v/v) solution of glacial acetic acid in methanol) to 10.0 mL of the stock solution and dilution to 25.0 mL with a 5 % (v/v) solution of glacial acetic acid in methanol. For the compensation solution, 10.0 mL of the stock solution were diluted to 25.0 mL with a 5 % (v/v) solution of glacial acetic acid in methanol. Finally, the absorbance of the test solution at 425 nm was measured after 30 min by comparison with the compensation solution.

The content of flavonoids was calculated as isoquercitrin, taking the specific absorbance of isoquercitrin to be 500, and represents the mean ± standard deviation of three independent experiments. This method yielded a **total flavonoid content of 133 ± 4 µg/mL (n=3) calculated as isoquercitrin**.

**Table S1. Proposed structure and MS data of the secondary plant metabolites identified in the *Equisetum arvense* extract.**

| **#** | **Proposed Structure^a^** | **[M‑H]^-^** | **Main Fragment Ions (>10% Rel. Int.)** | **References** |
| --- | --- | --- | --- | --- |
| 1 | Monocaffeoyl-tartaric acid isomer | 311.1 | MS^2^ [311.1]: 179.3, 149.3 | [DOI: 10.1016/0031-9422(94)00658-G](http://dx.doi.org/10.1016/0031-9422(94)00658-G) |
| 2 | Monocaffeoyl-tartaric acid isomer | 311.1 | MS^2^ [311.1]: 179.2, 149.3 | [DOI: 10.1016/0031-9422(94)00658-G](http://dx.doi.org/10.1016/0031-9422(94)00658-G) |
| 3 | Monoferuloyl-tartaric acid isomer | 325.1 | MS^2^ [325.1]: 193.2  MS^3^ [193.2]: 178.2, 149.9, 134.3, 133.8 | [DOI: 10.1016/j.foodchem.2010.05.017](http://dx.doi.org/10.1016/j.foodchem.2010.05.017) |
| 4 | Kaempferol-3-*O*-sophoroside-7-*O*-glucoside | 771.1 | MS^2^ [771.1]: 609.3  MS^3^ [609.3]: 429.3, 327.3, 285.2, 284.3, 255.2 | [DOI: 10.1016/0031-9422(94)00658-G](http://dx.doi.org/10.1016/0031-9422(94)00658-G)  [Europe PMC: CBA 347281](http://europepmc.org/abstract/CBA/347281) |
| 5 | Quercetin-3,7-di-*O*-glucoside | 625.1 | MS^2^ [625.1]: 463.3, 462.3, 301.3, 300.2, 299.3  MS^3^ [463.3]: 301.3, 300.2, 299.2, 271.2  MS^3^ [301.3]: 272.1, 271.1, 255.2, 151.1 | [DOI: 10.1016/0031-9422(94)00658-G](http://dx.doi.org/10.1016/0031-9422(94)00658-G) |
| 6 | Caffeoyl-malic acid | 295.1 | MS^2^ [295.1]: 179.2, 135.3, 133.3 | [DOI: 10.1016/j.foodchem.2007.11.032](http://dx.doi.org/10.1016/j.foodchem.2007.11.032)  [DOI: 10.1021/jf071314+](http://dx.doi.org/10.1021/jf071314+) |
| 7 | Monoferuloyl-tartaric acid isomer | 325.1 | MS^2^ [325.1]: 193.2  MS^3^ [193.2]: 149.5, 134.5 | [DOI: 10.1016/j.foodchem.2010.05.017](http://dx.doi.org/10.1016/j.foodchem.2010.05.017) |
| 8 | Kaempferol-3,7-di-*O*-glucoside | 609.0 | MS^2^ [609.0]: 489.3, 447.3, 285.3  MS^3^ [447.3]: 285.2, 284.3, 255.2  MS^4^ [284.3]: 255.2, 227.1 | [DOI: 10.1016/0031-9422(94)00658-G](http://dx.doi.org/10.1016/0031-9422(94)00658-G) |
| 9 | Kaempferol-3-*O*-rutinoside-7-*O*-glucoside | 755.1 | MS^2^ [755.1]: 593.3  MS^3^ [593.3]: 285.2  MS^4^ [285.2]: 267.2, 257.2, 256.1, 255.0, 241.2, 229.3, 227.3, 195.2, 189.1, 185.3, 173.2, 163.2, 151.2 | [DOI: 10.1016/0031-9422(94)00658-G](http://dx.doi.org/10.1016/0031-9422(94)00658-G) |
| 10 | Quercetin-3-*O*-sophoroside | 625.0 | MS^2^ [625.0]: 301.2, 300.2, 271.2 | [DOI: 10.1016/0031-9422(94)00658-G](http://dx.doi.org/10.1016/0031-9422(94)00658-G) |
| 11 | 4-Coumaric acid | 163.1 | MS^2^ [163.1]: 119.5 | [DOI: 10.1007/BF00571226](http://dx.doi.org/10.1007/BF00571226) |
| 12 | Kaempferol-3-*O*-sophoroside | 609.0 | MS^2^ [609.0]: 429.3, 285.2, 284.2, 255.2 | [DOI: 10.1016/0031-9422(94)00658-G](http://dx.doi.org/10.1016/0031-9422(94)00658-G) |
| 13 | Caffeic acid methyl ester^b^ | 193.1 | MS^2^ [193.1]: 178.2, 149.2, 134.3, 116.0 | [DOI: 10.1016/0031-9422(94)00658-G](http://dx.doi.org/10.1016/0031-9422(94)00658-G) |
| 14 | Protogenkwanin-4’-*O*-glucoside^c^ | 463.1 | MS^2^ [509.0]: 463.3, 301.3, 283.3  MS^3^ [283.3]: 268.2 | [DOI: 10.1016/0031-9422(94)00658-G](http://dx.doi.org/10.1016/0031-9422(94)00658-G) |
| 15 | Quercetin-3-*O*-glucoside^d^ | 463.0 | MS^2^ [463.0]: 301.2, 300.3  MS^3^ [301.2]: 271.2, 257.2, 256.2, 255.2, 254.2, 245.2, 193.2, 179.2, 151.2 | [DOI: 10.1016/0031-9422(94)00658-G](http://dx.doi.org/10.1016/0031-9422(94)00658-G) |
| 16 | Apigenin-*O*-glucoside | 431.1 | MS^2^ [431.1]: 269.2 | [DOI: 10.1016/0031-9422(94)00658-G](http://dx.doi.org/10.1016/0031-9422(94)00658-G) |
| 17 | Kaempferol-3-*O*-glucoside^d^ | 447.0 | MS^2^ [447.0]: 285.2, 284.2, 255.2  MS^3^ [285.2]: 255.2, 241.2, 240.1, 229.3, 227.2, 211.3 | [DOI: 10.1016/0031-9422(94)00658-G](http://dx.doi.org/10.1016/0031-9422(94)00658-G) |
| 18 | Dicaffeoyl-tartaric acid | 473.0 | MS^2^ [473.0]: 311.3, 293.2, 179.2, 149.2  MS^3^ [311.3]: 179.2, 149.2, 135.4  MS^4^ [149.2]: 131.2 | [DOI: 10.1016/0031-9422(94)00658-G](http://dx.doi.org/10.1016/0031-9422(94)00658-G) |
| 19 | Genkwanin-*O*-glucoside | 445.1 | MS^2^ [445.1]: 283.3, 268.3  MS^3^ [283.3]: 268.2 | [DOI: 10.1016/0031-9422(94)00658-G](http://dx.doi.org/10.1016/0031-9422(94)00658-G) |

^a^ The tentative identification is mainly based on literature data on the occurrence of the given compound in *E. arvense* and comparison of the UV- and MS^n^-data with literature values. Furthermore, the aglycone part of some flavonoids was identified by spectrum matching to an in-house library containing the MS^n^ spectra of 57 reference flavonoids using ESI compass 1.3 for HCT (Bruker Daltonics, Bremen, Germany).

^b^ This compound was proven not be ferulic or isoferulic acid by comparison with reference compounds.

^c^ This compound was predominately detected as [M+Cl]^-^ ion at m/z 509.0, which was also selected as precursor ion for the MS^n^ spectra.

^d^ These constituents were confirmed by comparison of the retention times, UV- and MS^n^-spectra with reference compounds.
